# Downregulation of miR-137 Facilitates CD4+ T Cell Pyroptosis in Systemic Lupus Erythematosus via Stimulating AMPK Pathway

**DOI:** 10.1155/2023/1241774

**Published:** 2023-02-09

**Authors:** Aimin Gong, Long Mi, Fangzhi Wei, Yanping Zhuang, Yitian Song, Chengdan Pan, Xuan Zhang, Guiling Lin, Zhiquan Wu, Ying Liu

**Affiliations:** ^1^Department of Basic Theory of Traditional Chinese Medicine, College of Traditional Chinese Medicine, Hainan Medical University, Haikou, 571199 Hainan, China; ^2^Department of Radiology, The First Affiliated Hospital of Hainan Medical University, Hainan Medical University, Haikou, 570100 Hainan, China; ^3^Academic Affairs Office, The Second Affiliated Hospital of Hainan Medical University, Haikou, 570311 Hainan, China; ^4^Department of Rehabilitation Medicine, Sanya Central Hospital, Sanya, 572000 Hainan, China; ^5^Department of Rheumatology and Immunology, Hainan General Hospital, Hainan Affiliated Hospital of Hainan Medical University, Haikou, 570311 Hainan, China

## Abstract

**Objective:**

From the pathogenic mechanism point of view, systemic lupus erythematosus (SLE) features prominently in T lymphocyte apoptosis. Yet the regulatory mechanism underlying SLE cell apoptosis remains to be explored. This research intends to clarify the role played by miR-137 in SLE and the underlying mechanisms.

**Methods:**

Twenty SLE patients (SLE group) and twenty healthy controls (control group) were selected, from whom peripheral blood CD4+ T cells were isolated via magnetic-activated cell sorting. Reverse transcription-polymerase chain reaction (RT-PCR) quantified miR-137 and AMP-activated protein kinase (AMPK) in CD4+ T cells. Further, transfection of miR-137 mimics and inhibitors into CD4+ T cells was carried out to alter miR levels. Levels of pyroptosis, apoptosis, and inflammatory- and pyroptosis-related proteins were determined through PI staining, flow cytometry, and Western blotting, respectively. A luciferase reporter gene assay identified the targeting relation between miR-137 and AMPK.

**Results:**

SLE patients showed downregulated miR-137 and upregulated AMPK in CD4+ T cells than controls. miR-137 upregulation by miR-137 mimic transfection inhibited Jurkat cell pyroptosis and apoptosis at both mRNA and protein levels and suppressed NOD-like receptor thermal protein domain-associated protein 3 (NLRP3) inflammasome activity and pyroptosis-related protein gasdermin D (GSDMD), while miR-137 inhibitor transfection contributed to completely opposite effects. miR-137 directly targeted AMPK, as indicated by the luciferase reporter gene assay. Furthermore, miR-137 inhibitor intervention induced healthy CD4+ T cell pyroptosis and apoptosis via mediating AMPK, whereas miR-137 mimic transfection into CD4+ T cells of SLE patients leads to opposite results.

**Conclusion:**

Upregulating miR-137 inhibits CD4+ T cell pyroptosis in SLE patients by modulating the AMPK pathway, suggesting the potential diagnostic and therapeutic role of miR-137 in SLE.

## 1. Introduction

Systemic lupus erythematosus (SLE), a chronic autoimmune-mediated inflammatory condition of connective tissue, has diverse clinical presentations ranging from mild skin disorders to catastrophic organ failure and obstetric complications, which can be life threatening in severe cases [1–3]. Reports on the incidence and prevalence of SLE over the past five years have revealed considerable variations across global regions and even subpopulations [4–6]. SLE is one of the prime reasons for death among young women. In a metastudy involving more than 26,000 affected women in the United States, these female SLE patients were found to have a 2.6-fold elevated all-cause mortality versus the general population, as well as a standardized mortality ratio (SMR) from cardiovascular disease rate of more than 2 times, a possibility of developing infection of more than 5 times and kidney disease of more than 7 times [7].

Until now, the specific pathogenesis of the disease has not been fully understood. But the dysimmunity caused by CD4+ T lymphocyte dysfunction, which is essential in pathogenic autoantibody production, is known to be an important inducement [8, 9]. CD4+ T cells, as a major T lymphocyte subpopulation, help B lymphocytes produce antibodies and activate macrophages, in addition to assisting the induction of cellular and humoral immunity [10]. Following activation, CD4+ T cells release cytokines in large quantities and modulate the activities of T and B lymphocytes, monocytes/macrophages, and other cells, which in turn leads to the generation of massive autoantibodies in the body, resulting in multisystem damage [11]. The formation of autoantibodies is considered to be the result of altered pathways of cell death, including apoptosis, necrosis, formation of neutrophil extracellular traps (NETs), and increased low-density granulocyte production [12–14]. Pyroptosis, like apoptotic programmed cell death, is actually a proinflammatory programmed cell death that is featured by inflammatory intracellular substance release and cell membrane rupture [15, 16]. In a recent pioneering study [17], pyroptosis was found to be the primary reason for CD4+ T cell death from nonproductive human immunodeficiency virus type 1 (HIV-1) infection, indicating that pyroptosis-induced inflammation, CD4+ T cell death, and immune system activation constitute a unifying theme of the immunopathogenesis of HIV infection. The role played by pyroptosis in in vivo CD4+ T cell death, particularly during early-stage infection, however, has not yet been characterized.

Belonging to small noncoding RNAs, microribose nucleic acids (miRNAs) modulate gene levels through acting on their target mRNAs, triggering translation suppression or mRNA degradation [18]. It has been found that miRNAs are critical in immune homeostasis and participate in immune cell development and dysfunction by acting on the posttranscriptional level of genes, which are strongly linked to autoimmune diseases (AIDs) [19, 20]. A study found that inhibiting miRNA-448 hinders CD4+ T cell inflammatory activation by upregulating SOCS5, a SLE cytokine [21]. Malregulated expression of miR-137 has been reported in some studies to cause inflammation and AIDs [22, 23]. Yet the function of serum miR-137 in SLE diagnosis and treatment and CD4+ T cell apoptosis in SLE patients has not been reported. And as a cellular signal converter and energy sensor, AMP-activated protein kinase (AMPK) is under the regulation of various metabolic stresses [24]. Activation of AMPK is usually accompanied by the increase of catabolism and the decrease of anabolism realized through substrate phosphorylation [25, 26]. Recent studies have found a connection between AMPK activation and anti-inflammatory reaction [27, 28]. Intentionally activating AMPK has been proposed as a strategy to treat hyperglycemia/hyperlipidemia-, redox stress-, or inflammation-associated metabolic dysregulation [29–31]. The favorable anti-inflammatory and immunosuppressive action of multiple AMPK activators against inflammatory diseases and AIDs have been demonstrated in various cell and preclinical models [32, 33]. Besides, the interaction between AMPK and inflammation has been highlighted, pointing out that AMPK activation reduces NF-*κ*B-induced inflammation and immunoreaction (through decreasing proinflammatory cytokine release and impairing Th1 and Th17 cell differentiation) [34]. Through several downstream targets of AMPK, AMPK activators (metformin (MET), AICAR, etc.) have been shown to indirectly weaken NF-*κ*B signals and alleviate inflammation [35]. Considering that metabolic control is at the core of mitigating inflammation, it is reasonable to resolve inflammation through AMPK activation. Moreover, miR-137 has been reported to inhibit TCF4 in a targeted way and reverse osteoarthritis progression through AMPK/NF-*κ*B axis [36]. Therefore, in this study, we determined miR-137 in SLE patients' CD4+ T cells and discussed its potential role and the mechanism in regulating SLE cell apoptosis and dysfunction. At the same time, we found by verification that AMPK may be miR-137's direct target gene, which helps it regulate cell death through inducing pyroptosis.

## 2. Data and Methods

### 2.1. Clinical Data

Twenty SLE patients (5 males and 15 females, age range: 34.6 ± 3.7 years) hospitalized between June 2019 and June 2021, all of whom met the SLE classification standard revised by the Systemic Lupus International Collaboration Clinics (SLICC) in 2009, were collected. The control group had a total of 20 healthy people (male:female: 9 : 11) aged 34.1 ± 2.8 years, with normal antinuclear antibody, antinuclear antibody spectrum, erythrocyte sedimentation rate (ESR), blood routine, urine routine, and immunoglobulin examination results. Cases and controls did not show statistical differences in age and sex composition ratio. Informed consent was obtained from the research subjects prior to the study, and this research was approved by our hospital's Ethics Committee.

### 2.2. Determination of Peripheral Blood CD4+ T Cells and Monocyte Isolation

Five to ten milliliters of EDTA anticoagulated venous whole blood were sampled from patients and health controls participating in the study, 100 *μ*L of which was put into a special flow test tube and added with 10 *μ*L of FITC anti-CD4 antibody for 15 min of incubation in the dark. Then, 2 mL of tenfold diluted hemolysin was added for 10 min, followed by 5 min of centrifugation (1,500 r/min). After supernatant removal, it was mixed with 1 mL of PBS and centrifuged again (1500 r/min) for 5 min, followed by another supernatant removal and mixing with 400 *μ*L PBS, for flow cytometry (FCM) determination of the CD4+ T cell percentage. Another anticoagulated whole blood sample was prepared, and lymphocytes were separated by gradient centrifugation with human peripheral blood mononuclear cell (PBMC) separation medium (Beijing Dakewe Biotech, China) according to the instructions.

### 2.3. Cell Isolation and Culture

The isolated PBMCs were used to sort CD4+ T cells by using a CD4+ T cell immunomagnetic bead separation kit (Takara, Japan). PBMCs were centrifuged (1000 r/min) for 10 min after the addition of 20 *μ*L magnetic bead mixture and 30 min of cultivation (4°C). The PBMCs were then taken out into a resuspension buffer and added with the magnetic bead sorting medium, followed by 3 washes with the buffer to remove the magnetic field and treatment with the eluting buffer to obtain CD4+ T cells that were then placed to an OpTmizer CTS T-Cell Expansion medium (Gibco, USA) added with 1% L-glutamine+1% penicillin/streptomycin. Jurkat cells were placed in an RPMI 1640 medium where fetal bovine serum (15%) and penicillin/streptomycin (1%) were added. All cells were placed in a 37°C and 5% CO_2_ incubator for routine culture.

### 2.4. Cell Transfection

100 nM each of mimic, inhibitor, and mimic/inhibitor control of miR-137 was transiently transfected into Jurkat cells and CD4+ T cells with the Lipofectamine RNAiMAX transfection reagent (Invitrogen) as per the supplier's recommendations and collected 48 h later for further analysis. GenePharma Co., Ltd., Shanghai, China, was entrusted to chemically synthesize miR-137 mimic (5′-GAUGCGCAUAAGAAUUCGUUAUU-3′), mimic control (5′-UCGCUUGGUGCAGGUCGGGAA-3′), miR-137 inhibitor (5′-CUACGCGUAUUCUUAAGCAAUAA-3′), and inhibitor NC (5′-GGUUCGUACGUACACUGUUCA-3′).

### 2.5. qRT-PCR

TRIzol reagent ordered from Invitrogen was responsible for cell total RNA extraction, after which reverse transcription into cDNA was carried out with the use of a PrimeScript RT Master Mix purchased from Takara, Japan. Using this cDNA as a template, PCR was performed on ABI PRISM 7300 sequence detection system (Applied Biosystems Inc.) as instructed by the SYBR Premix Ex Taq II kit manuals (Takara). Specific primers used in qRT-PCR, which were presented as below, were synthesized by Shanghai Sangon Biotech: miR-137: sense (5′-3′): CGCGTAGTCGAGGAGAGTACCA; antisense (5′-3′): AGTGCAGGGTCCGAGGTATT; U6: sense: CTCGCTTCGGCAGCACA; antisense: AACGCTTCACGAATTTGCGT; AMPK: sense: TTTGCGTGTACGAAGGAAGAAT; antisense: CTCTGTGGAGTAGCAGTCCCT; *β*-actin: sense: CCTGGCACCCAGCACAAT; and antisense: GGGCCGGACTCGTCATAC. 2^−ΔΔCt^ was used to calculate miR-137 and AMPK expression relative to U6 and *β*-actin, respectively. Mean values were obtained after three repeated measurements.

### 2.6. Western Blotting Experiment

RIPA lysate isolated protein isolation from cells as instructed by the manufacturer's manuals, after which the bicinchoninic acid (BCA) method was employed for protein content determination. The proteins were transferred to a membrane made of polyvinylidene fluoride (PVDF) after sodium dodecyl sulfate-polyacrylamide gel electrophoresis (SDS-PAGE; 12%) and blocked indoor for 1 h with 5% defatted milk, followed by a night-long incubation (4°C) by immersing in primary antibodies (Cell Signaling Technology), p-AMPK (Cell Signaling Technology), NLRP3 (Cell Signaling Technology), GSDMD (Cell Signaling Technology), ASC (R&D Systems), pro-caspase-1 (R&D Systems), caspase-1 p20 (R&D Systems), pro-IL-1*β* (Abcam), IL-1*β* (Abcam), pro-IL-18 (Abcam), IL-18 (Abcam), and *β*-actin (Abcam) all diluted at 1 : 1000. The HRP secondary antibody diluted at 1 : 1000 was then added for 1 h of indoor cultivation. Finally, the protein bands were exposed in MultiImager, and protein levels were calculated according to the bands' gray values with *β*-actin as internal reference.

### 2.7. Double Luciferase Reporter (DLR) Assay

AMPK mRNA 3′-untranslated region (3′-UTR) and its site-directed mutant were subjected to PCR amplification for cloning into the psiCHECK-2 vector (Promega). Subsequently, wild-type (WT) or mutant (MUT) luciferase plasmid was cotransfected into 293 T with miR-137 mimetic/negative control. Renin and firefly luciferase activities, with Renin luciferase activity as standardized index, were determined by the DLR system (Promega).

### 2.8. Hoechst 3342/Prodium Iodide (PI) Staining

Pyroptosis-induced cell membrane damage was assessed through Hoechst 3342/PI dual staining. In brief, cells were cultured in the wells of 24-well plates and immersed in the dark in a medium mixed with Hoechst 33342 and PI fluorescent dyes (both from Sigma, USA) for staining at room temperature. Twenty minutes after staining, cells were observed microscopically to determine PI positive cell percentage.

### 2.9. Apoptosis

The adherent cells were gathered, and apoptosis was determined following the operating instructions of the Annexin V-FITC/7-AAD Apoptosis kit (BD Bioscience): after 15 min of light-tight cultivation with Annexin V-FITC staining solution (5 *μ*L), they were gently mixed with 5 *μ*L of 7-AAD staining solution for 5 min of incubation at 2-8°C in the dark, followed by FCM determination of cell apoptosis.

#### 2.9.1. Statistical Analysis

GraphPad Prism software statistically analyzed the data. Each test ran at least in triplicates. All experimental data of each group were given mean ± standard deviation, and the mean was pairwisely compared with Student's *t*-test. A *P* value < 0.05 was deemed significant for all tests.

## 3. Results

### 3.1. miR-137 and AMPK Levels in Isolated CD4+ T Cells

**Figure 1:**
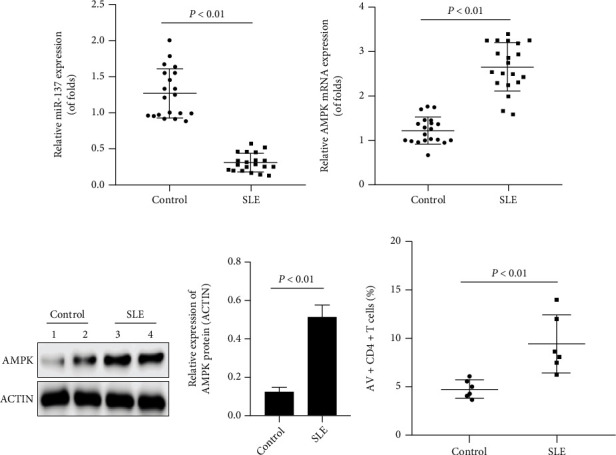
miR-137 and AMPK expressions in isolated CD4+ T cells. (a) miR-137 in isolated CD4+ T cells; (b) AMPK mRNA in isolated CD4+ T cells; (c) AMPK protein in isolated CD4+ T cells; (d) apoptosis level by flow cytometry. Each test ran in triplicates. The means were pairwisely compared using Student's *t*-test. *P* < 0.01 means the presence of statistical significance.

qRT-PCR quantified endogenous miR-137 expression in CD4+ T cell specimens separated from 20 SLE cases and 20 healthy donors. The results revealed obviously lower miR-137 (Figure 1(a)) and higher AMPK expression (Figures 1(b) and 1(c)) in CD4+ T cells of cases versus controls. And according to Annexin V/PI dual staining, the percentage of apoptosis of freshly isolated CD4+ T cells was markedly higher in cases versus controls (Figure 1(d)).

### 3.2. miR-137 Induces Cell Pyroptosis through AMPK

**Figure 2:**
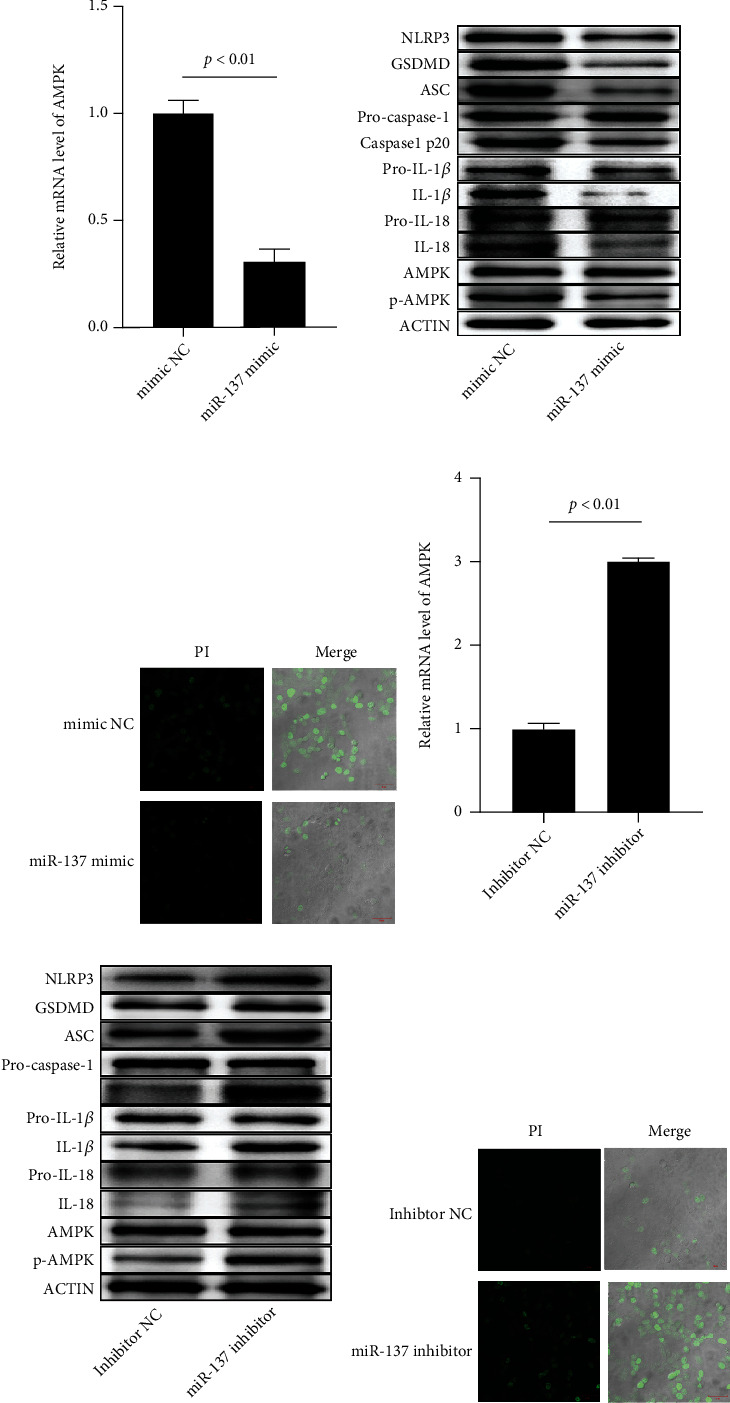
miR-137 regulates AMPK and induces pyroptosis. (a) Impact of miR-137 mimic transfection on AMPK mRNA expression. (b) Impact of miR-137 mimic transfection on inflammation and pyroptosis-related proteins. (c) Impact of miR-137 mimic transfection on cell pyroptosis; (d) AMPK mRNA expression after miR-137 inhibitor transfection. (e) Related protein expression after miR-137 inhibitor transfection. (f) Impact of miR-137 inhibitor transfection on cell pyroptosis. Each test ran in triplicates. The means were pairwisely compared using Student's *t*-test. *P* < 0.01 means the presence of statistical significance.

To determine the modulation of miR-137 on AMPK, we investigated the role played by miR-137 through transfecting miR-137 mimic or inhibitor into Jurkat cells. According to qRT-PCR, AMPK expression was remarkably reduced by miR-137 mimic transfection at both mRNA and protein levels while was notably enhanced by miR-137 inhibitor transfection (Figures 2(a), 2(b), 2(d), and 2(e)). In addition, we studied the role played by miR-137 in regulating AMPK-mediated inflammatory pathway proteins and pyroptosis-related pathway proteins. NLRP3, GSDMD, ASD, caspase-1 p20, and IL-1*β* protein levels were found to be notably decreased by miR-137 mimic transfection while were statistically enhanced by miR-137 inhibitor transfection (Figures 2(b) and 2(e) and Supplementary Figure 1). Meanwhile, Hoechst 3342/PI staining showed that miR-137 mimic transfection kept the integrity of cell membrane caused by pyroptosis (Figures 2(c) and 2(f)).

### 3.3. miR-137 Directly Targets AMPK

**Figure 3:**
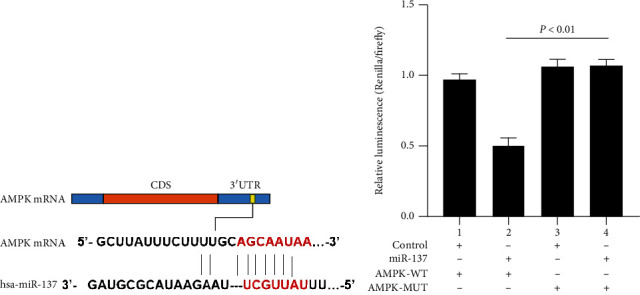
Targeted regulation of AMPK by miR-137. (a) Schematic diagram of miR-137's binding sites in AMPK 3′-UTR; (b) DLR gene assay. Each test ran in triplicates. The means were pairwisely compared using Student's *t*-test. *P* < 0.01 means the presence of statistical significance.

To demonstrate the direct interaction between the two genes, we performed a DLR assay. Through online prediction websites, we predicted the binding site of AMPK mRNA 3′-UTR (Figure 3(a)). Subsequently, the DLR gene assay verified that luciferase activity decreased under AMPK-WT+miR-137 mimic cotransfection (*P* < 0.01), but the fluorescence activity of other cotransfected combinations did not alter significantly (Figure 3(b)). The results suggest the ability of miR-137 to directly target AMPK.

### 3.4. miR-137 Inhibitor Induces Normal CD4+ T Cell Pyroptosis via Mediating AMPK

**Figure 4:**
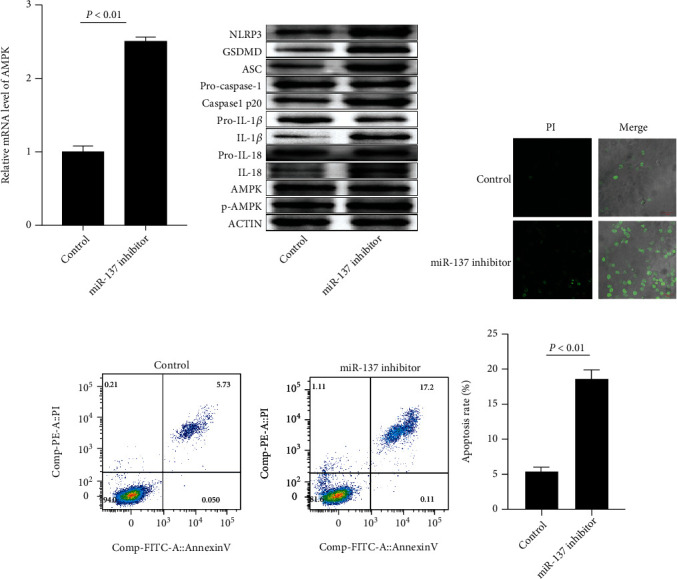
miR-137 inhibitor induces normal CD4+ T cell pyroptosis via mediating AMPK. (a) AMPK mRNA expression in normal CD4+ T cells after miR-137 inhibitor transfection. (b) Impact of miR-137 inhibitor transfection on inflammation and pyroptosis-related proteins in normal CD4+ T cells. (c) Impact of miR-137 inhibitor transfection on normal CD4+ T cell pyroptosis. (d) Impact of miR-137 inhibitor transfection on normal CD4+ T cell apoptosis. Each test ran in triplicates. The means were pairwisely compared using Student's *t*-test. *P* < 0.01 means the presence of statistical significance.

We assessed the impact of the miR-137 inhibitor on isolated healthy CD4+ T cells to further analyze the functional interaction of miR-137 with AMPK. miR-137 inhibitor transfection remarkably enhanced AMPK at both mRNA and protein levels in normal CD4+ T cells (Figure 4(a)), as well as AMPK-mediated inflammation and pyroptosis pathway-related protein expression (Figure 4(b) and Supplementary Figure 2). In addition, we used Hoechst 3342/PI staining and FCM to analyze whether miR-137 can regulate AMPK-mediated cell pyroptosis and apoptosis. As indicated by Figures 4(c) and 4(d), miR-137 inhibitor transfection induced normal CD4+ T cell pyroptosis and apoptosis.

### 3.5. miR-137 Mimic Inhibits CD4+ T Cell Pyroptosis by Mediating AMPK

**Figure 5:**
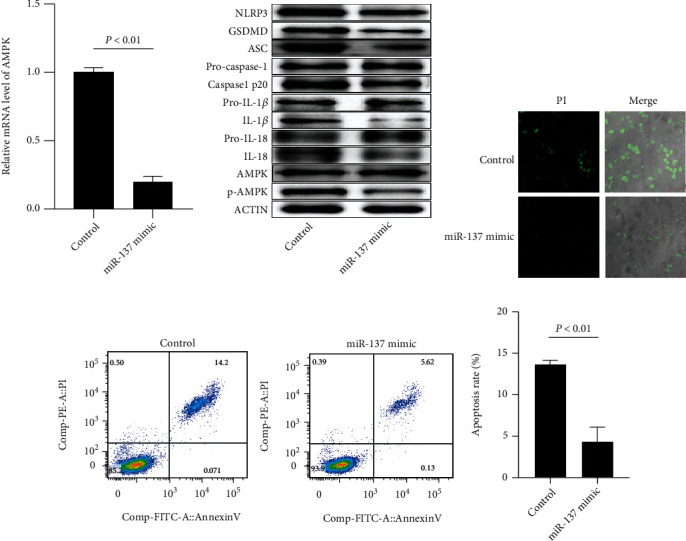
miR-137 mimic inhibits CD4+ T cell pyroptosis via mediating AMPK in SLE. (a) AMPK mRNA expression in SLE patients' CD4+ T cells after transfecting miR-137 mimic. (b) Inflammation and pyroptosis-related proteins in SLE patients' CD4+ T cells after transfecting miR-137 mimic. (c) Influence of miR-137 mimic transfection on SLE patients' CD4+ T cell pyroptosis. (d) Impact of miR-137 mimic transfection on SLE patients' CD4+ T cell apoptosis. Each test ran in triplicates. The means were pairwisely compared using Student's *t*-test. *P* < 0.01 means the presence of statistical significance.

As miR-137 was downregulated in SLE patients' CD4+ T cells, we studied its function in patients' CD4+ T cells by transfecting miR-137 mimic. miR-137 mimic transfection statistically reduced AMPK mRNA and protein expression in CD4+ T cells (Figure 5(a)), as well as AMPK-mediated inflammation and pyroptosis pathway-related protein expression (Figure 5(b) and Supplementary Figure 3). In addition, Hoechst 3342/PI staining and FCM showed that miR-137 mimic transfection protected CD4+ T cells against pyroptosis and apoptosis (Figures 5(c) and 5(d)).

## 4. Discussion

SLE is an AID in which autoreactive CD4+ T cells play a vital part and are active mediators of SLE pathogenesis, which rely on glycolysis to function as inflammatory effectors [37]. Pyroptosis can also cause excessive inflammatory damage to cells [38], while miRNAs, with wide involvement in regulating innate and adaptive immune cell differentiation, development, and function, are critical in biological development and physiological activities [39]. SLE is a disease with unknown pathogenesis and susceptibility to recurrence and remission, which brings great pain to patients. So, exploring abnormal expression molecules of CD4+ T lymphocytes in SLE patients may provide reliable targets for the diagnosis, efficacy, and prognosis evaluation of SLE and is also crucial for inhibiting SLE apoptosis and alleviating related cellular dysfunction.

Numerous studies have shown that the abnormal miRNA expression is involved in the nosogenesis of SLE [40, 41]. Along with the development of miRNA panels, miRNAs have been a research hotspot in the diagnostic area, but the therapeutic market that is driven primarily by antagomiR and miRNA mimic products is underdeveloped. Miravirsen [42] and RG-101 [43], produced by Roche/Santaris and Regulus Therapeutics, respectively, are developed for the treatment of hepatitis C and are believed to be flagship products for the disease in the future. Currently, all miRNA-based drugs are in clinical trials, yet none have made a breakthrough. With the implementation of several plans, the miRNA market will undoubtedly become a decisive factor in the coming years, even if it is still in its infancy.

The present research revealed underexpressed miR-137 and elevated AMPK in CD4+ T cells of SLE cases versus healthy donors. miRNAs participate in multiple biological processes, including immune system stabilization, cell growth, differentiation, and apoptosis. Evidence shows markedly increased miR-21 and evidently reduced miR-98 in SLE patients' CD4+ T cells versus the healthy population [44, 45]. This study was also the first to reveal a significant reduction in miR-137 in SLE patients' CD4+ T cells. Then, we intervened miR-137 to observe its effect on SLE cells. The results identified that transfection-induced miR-137 upregulation suppressed Jurkat and CD4+ T cell pyroptosis and apoptosis in SLE patients at both mRNA and protein levels while suppressing NLRP3 inflammasome activity and pyroptosis-related protein GSDMD expression. GSDMD has recently been identified as a key effector of pyroptosis, which functions by activating N-terminal insertion, oligomerization, and membrane pore formation following cleavage of inflammatory caspases [46, 47]. Under normal cell conditions, the C-terminal of GSDMD will automatically inhibit the pore-forming activity of the N-terminal [48]. When inflammasomes, such as NLRP3 inflammasome, are activated by extracellular signals associated with pyroptosis, they subsequently divide and activate caspase-1, -4, -5, and -11. Thus, the activated caspase-1 cleaves and isolates GSDMD N- and C-terminals [49]. In this study, caspase-1 p20 levels were also inhibited after miR-137 mimic transfection. Notably, MET has been indicated in previous studies to reduce cell pyroptosis via NLRP3-GSDMD axis, thus protecting against intestinal ischemia-reperfusion injury [50]. Also, in the study of Peng et al. [51], piperine ameliorated lupus nephritis progression by blocking AMPK activation in pristane-injected mice. And via targeting AMPK, piperine markedly reduced NLRP3 inflammasome activation and inhibited proinflammatory cytokine release, thus blocking tubular epithelial cell pyroptosis.

Meanwhile, we found that AMPK was also regulated when miR-137 expression was altered. Thus, miR-137 may act on SLE by mediating AMPK. Subsequently, we found that miR-137 can directly target AMPK through DLR gene assay. In addition, miR-137 inhibitor induced healthy CD4+ T cell pyroptosis and apoptosis via mediating AMPK, whereas miR-137 mimic transfection into CD4+ T cells of SLE patients led to opposite effects. AMPK, which is present in nearly all cells and tissues of mammals [52], is critical in modulating energy and substance metabolism and interferes with a range of biological functions (cell proliferation, apoptosis, inflammation, etc.) [53]. AMPK activators (MET and A769662) have been shown to lower mechanical hypersensitivity in mice and rats with neuropathic pain, revealing a potential mechanism for the treatment of neuropathic pain via AMPK [54].

However, this study still has room for improvement. First, we can investigate the correlation of miR-137 expression with clinicopathologic features of SLE patients and disease severity. Second, AMPK is not the only downstream target of miR-137 in SLE. As discussed in the introduction section, NF-*κ*B is also the key regulator of inflammation. Thus, studies on the potential response of the AMPK/NF-*κ*B axis to miR-137 can be supplemented to further reveal the related regulatory mechanisms.

## 5. Conclusion

miR-137 is essential in the pathogenesis of SLE. Downregulation of miR-137 results in dysregulated SLE cell apoptosis, in part by targeting AMPK for direct interaction, leading to abnormal T cell responses. Therefore, miR-137 may be a novel strategy for SLE treatment. In addition, theoretically, serum molecules are susceptible to environmental influence [55], while molecule expression in CD4+ T cells is less affected, showing greater advantages in evaluating the severity of SLE. Nonetheless, further research is warranted for confirmation.

## Data Availability

The labeled dataset used to support the findings of this study is available from the corresponding author upon request.

## Supplementary Materials

Supplementary 1 Supplementary Figure 1: (A) relative protein level after transfected with miR-137 mimic. (B) Relative protein level after transfected with miR-137 inhibitor.

Supplementary 2 Supplementary Figure 2: inflammation and pyroptosis-related protein levels in normal CD4+ T cells after transfected with miR-137 inhibitor.

Supplementary 3 Supplementary Figure 3: inflammation and pyroptosis-related protein levels in normal CD4+ T cells after transfected with miR-137 mimic.
