# Supplementary figures and images for: Downregulation of miR-137 Facilitates CD4+ T Cell Pyroptosis in Systemic Lupus Erythematosus via Stimulating AMPK Pathway
Source: J Immunol Res. 2023 Feb 9;2023:1241774. doi: 10.1155/2023/1241774 (PMC9936506; doi:10.1155/2023/1241774)

A

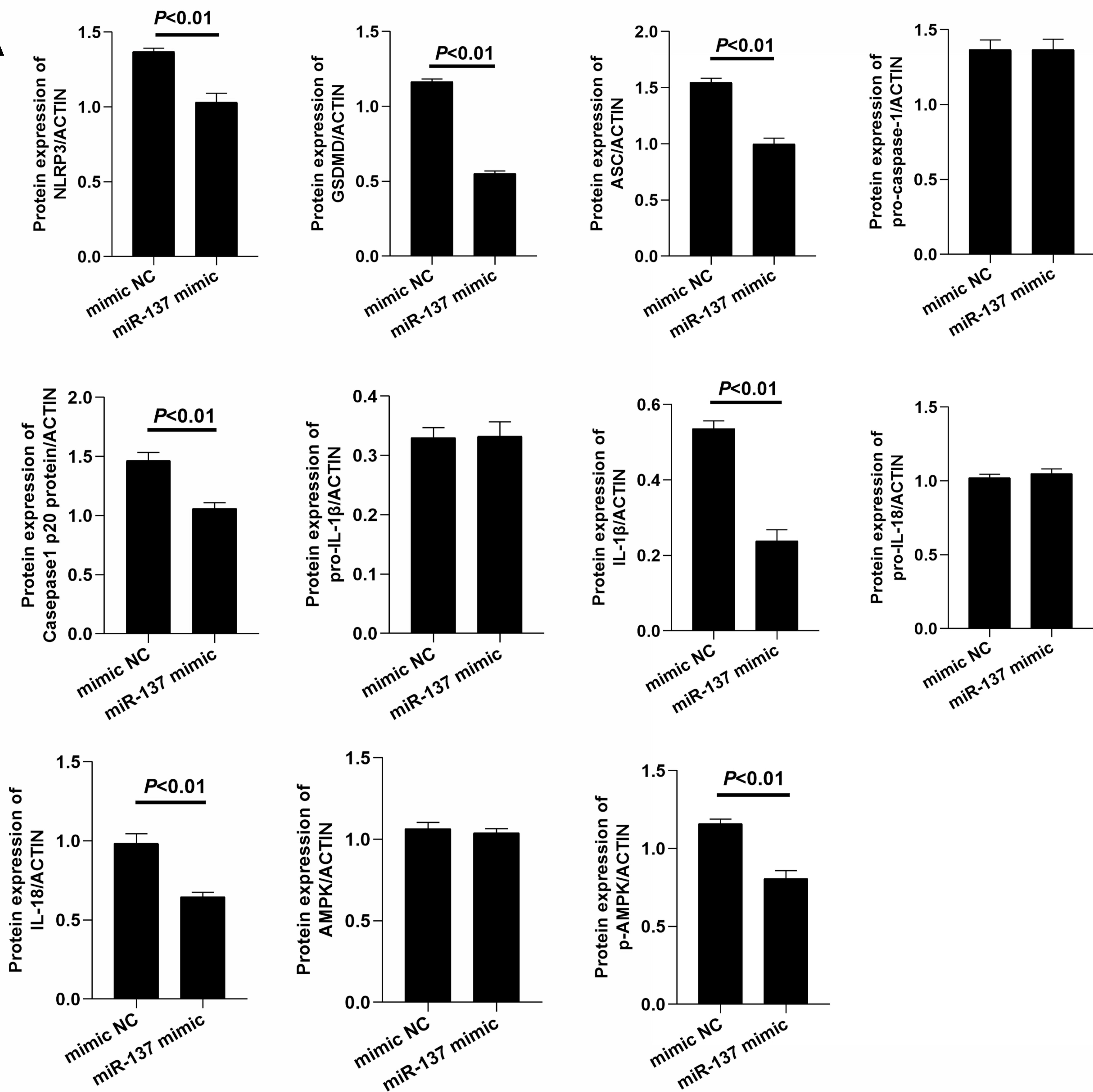

B

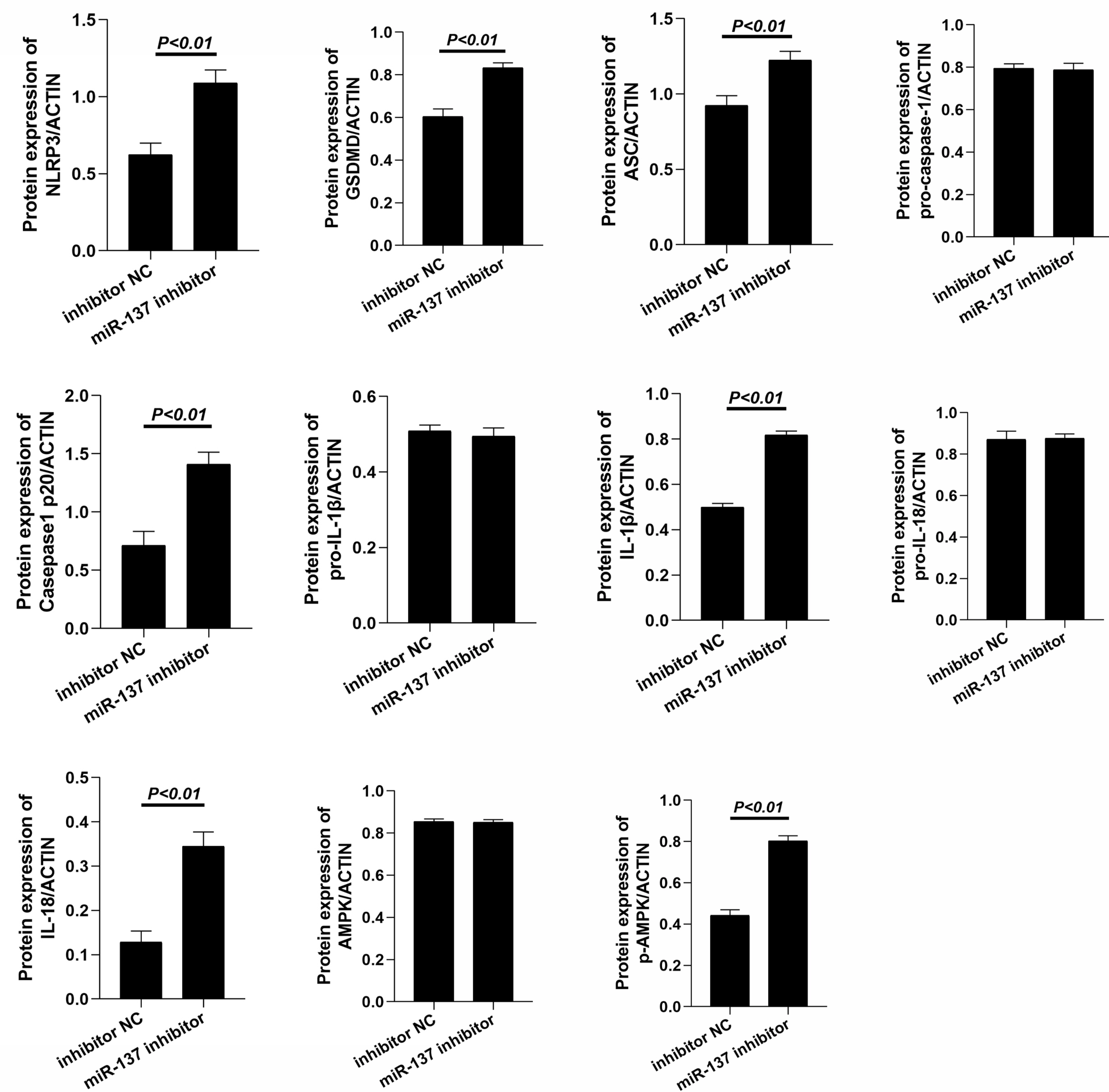

Supplement: Supplementary 1 — Supplementary Figure 1: (A) relative protein level after transfected with miR-137 mimic. (B) Relative protein level after transfected with miR-137 inhibitor. [file 1241774.f1.pdf]

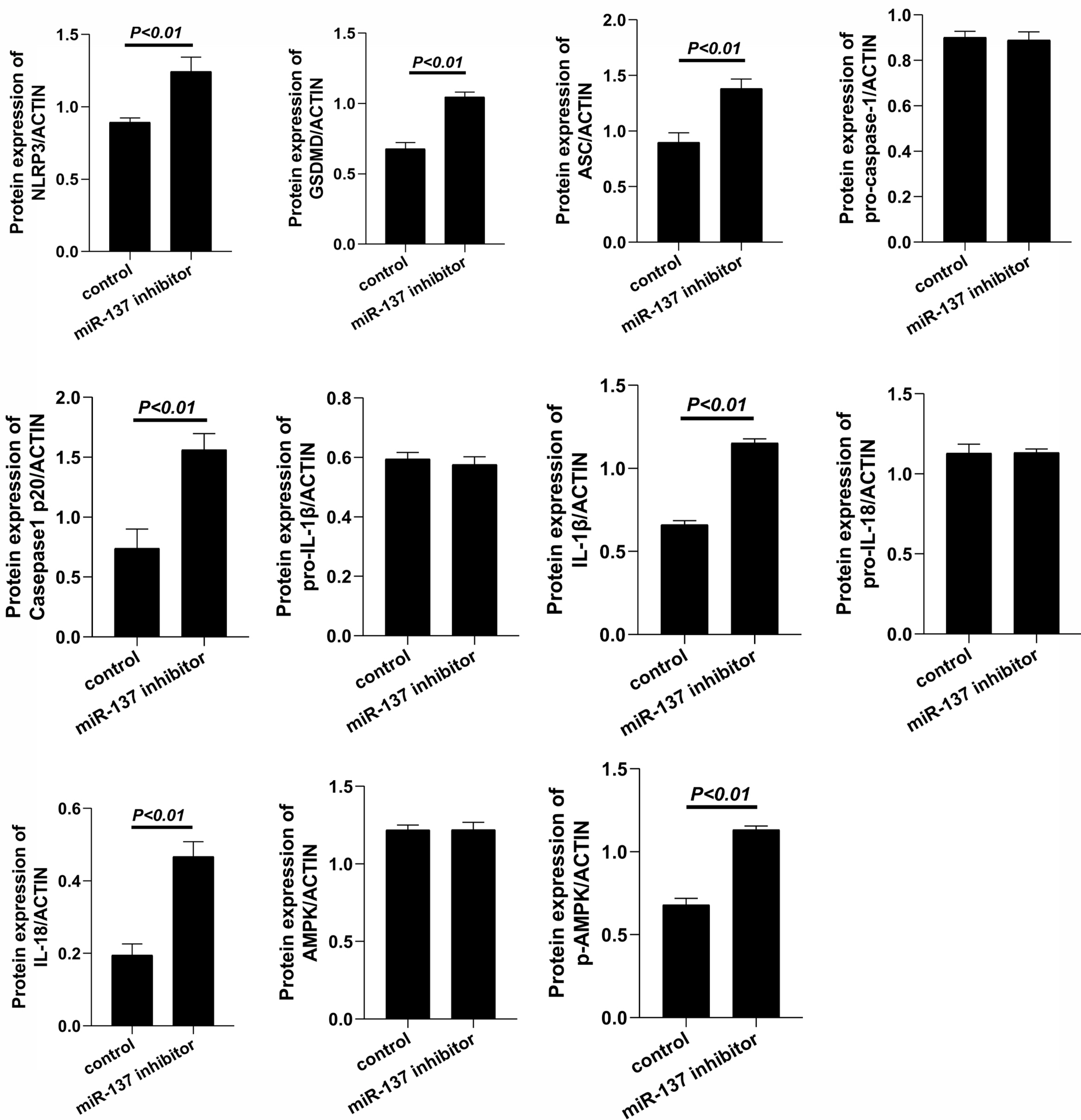

Supplement: Supplementary 2 — Supplementary Figure 2: inflammation and pyroptosis-related protein levels in normal CD4+ T cells after transfected with miR-137 inhibitor. [file 1241774.f2.pdf]

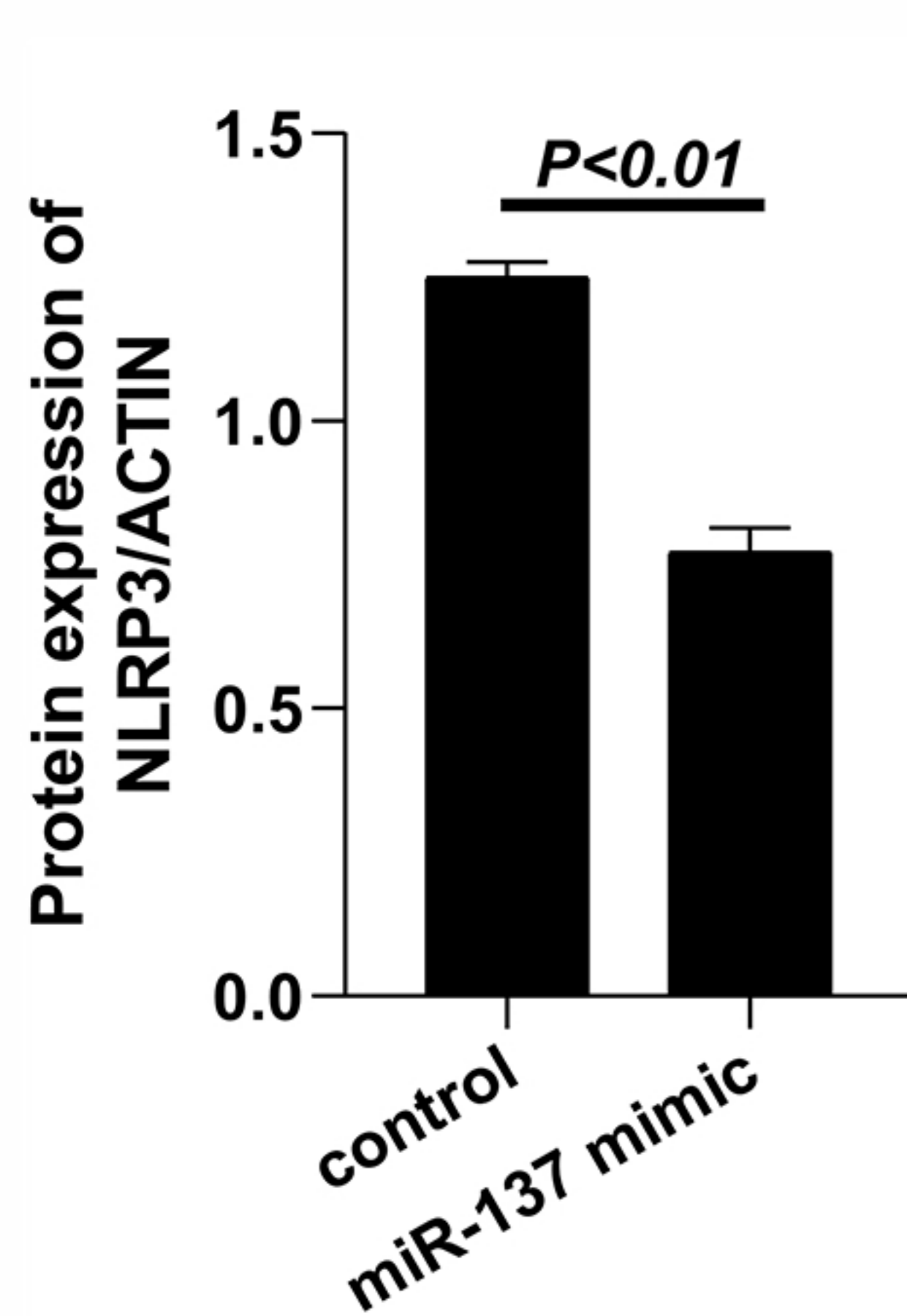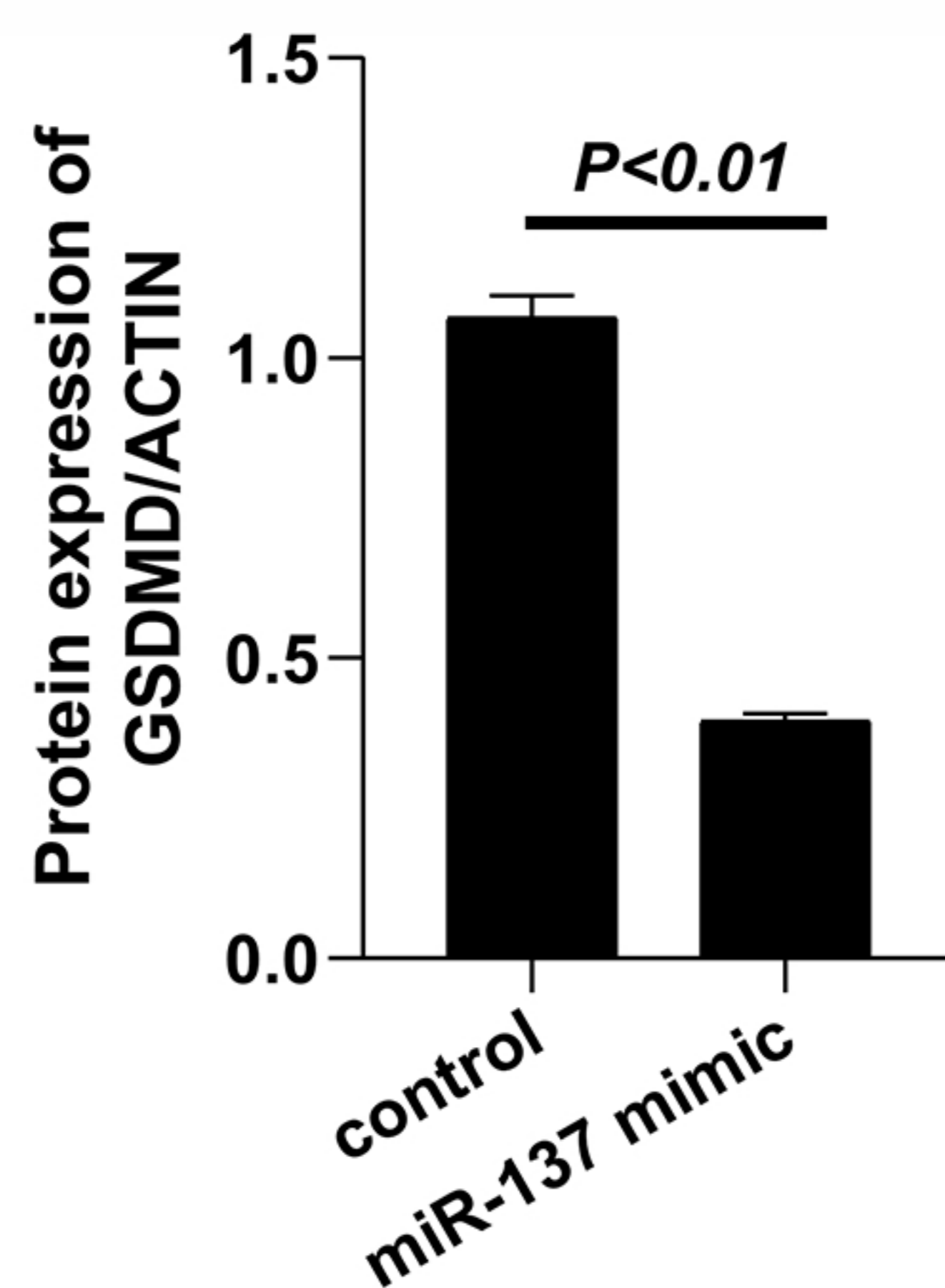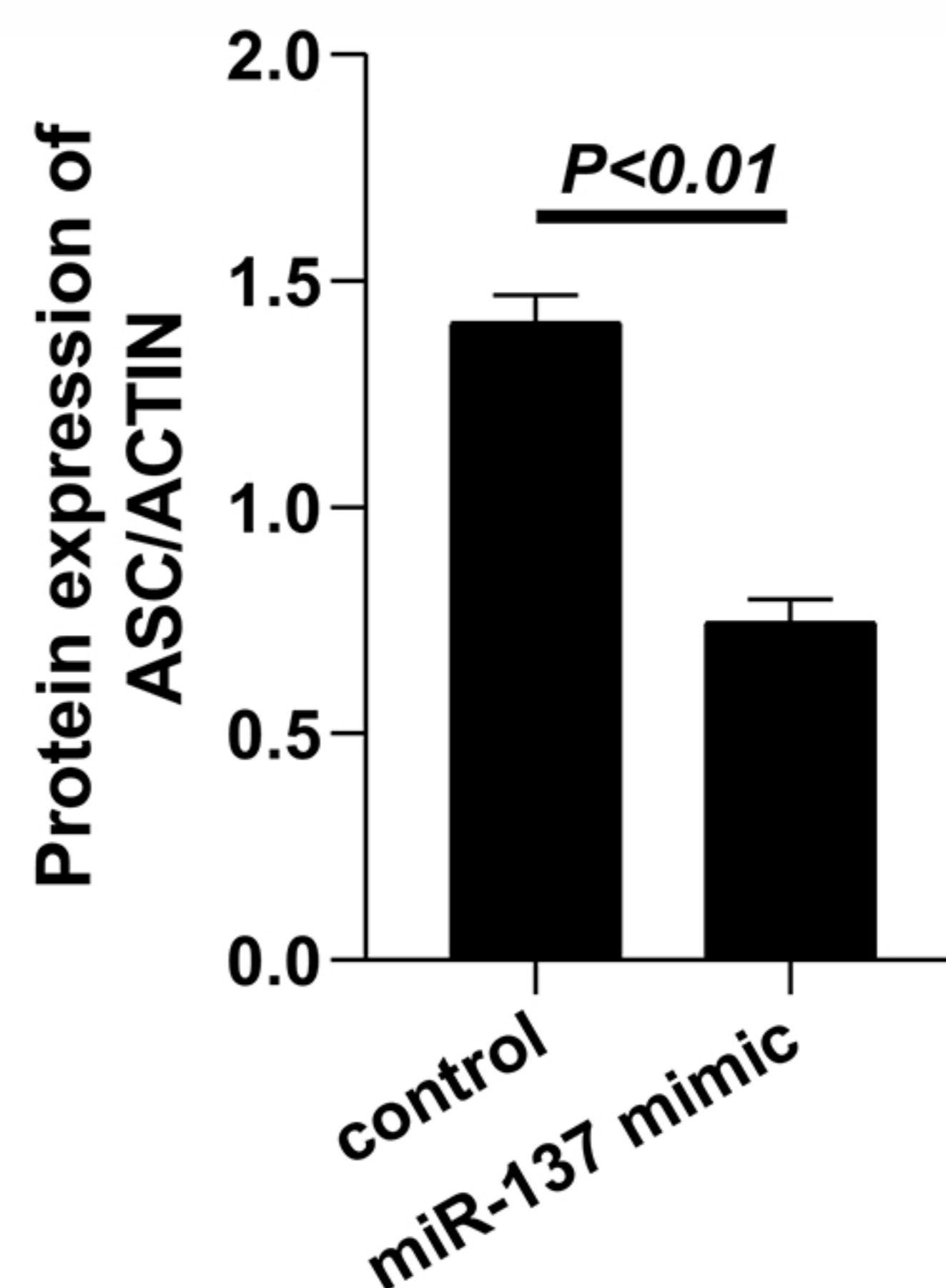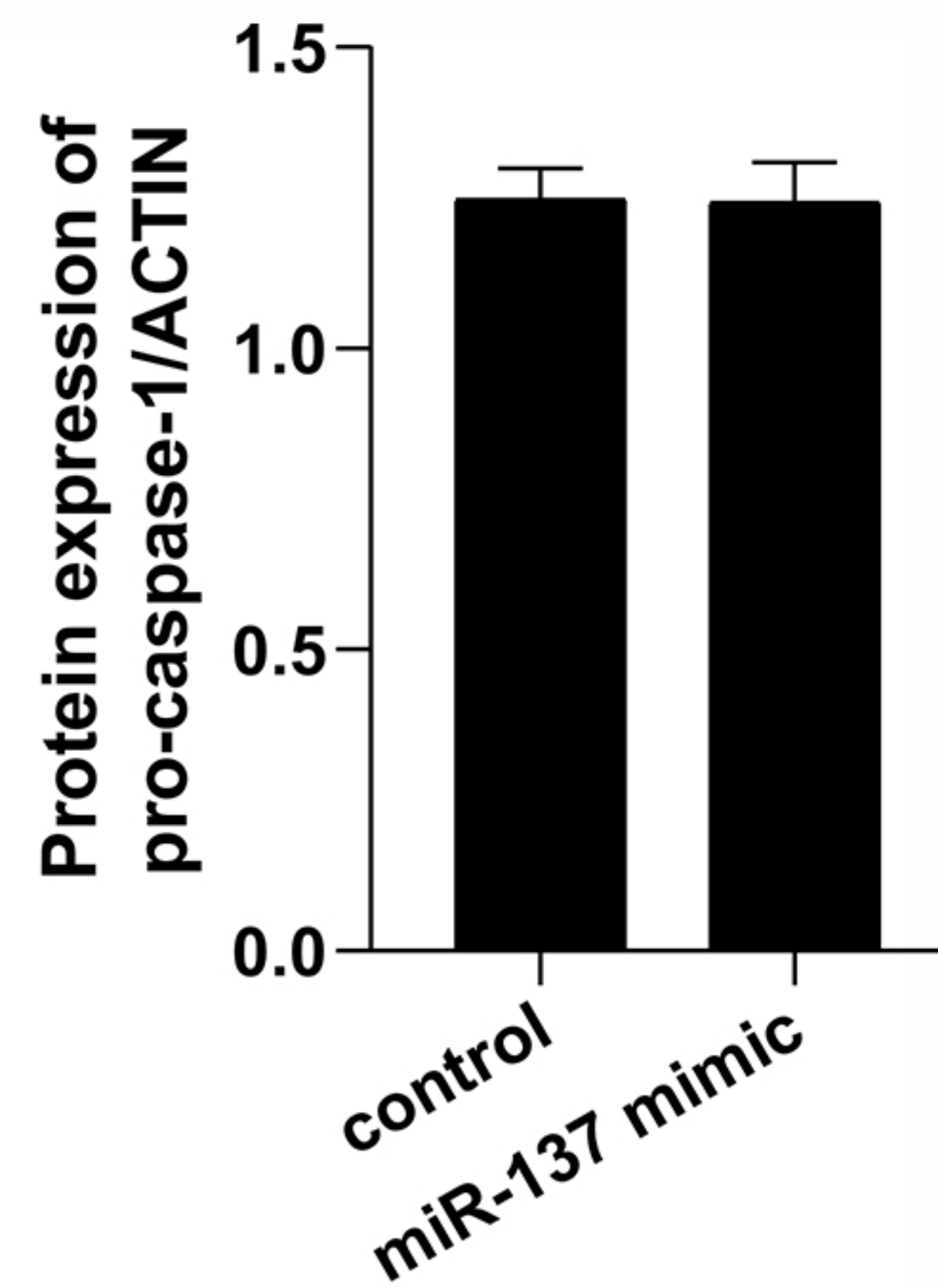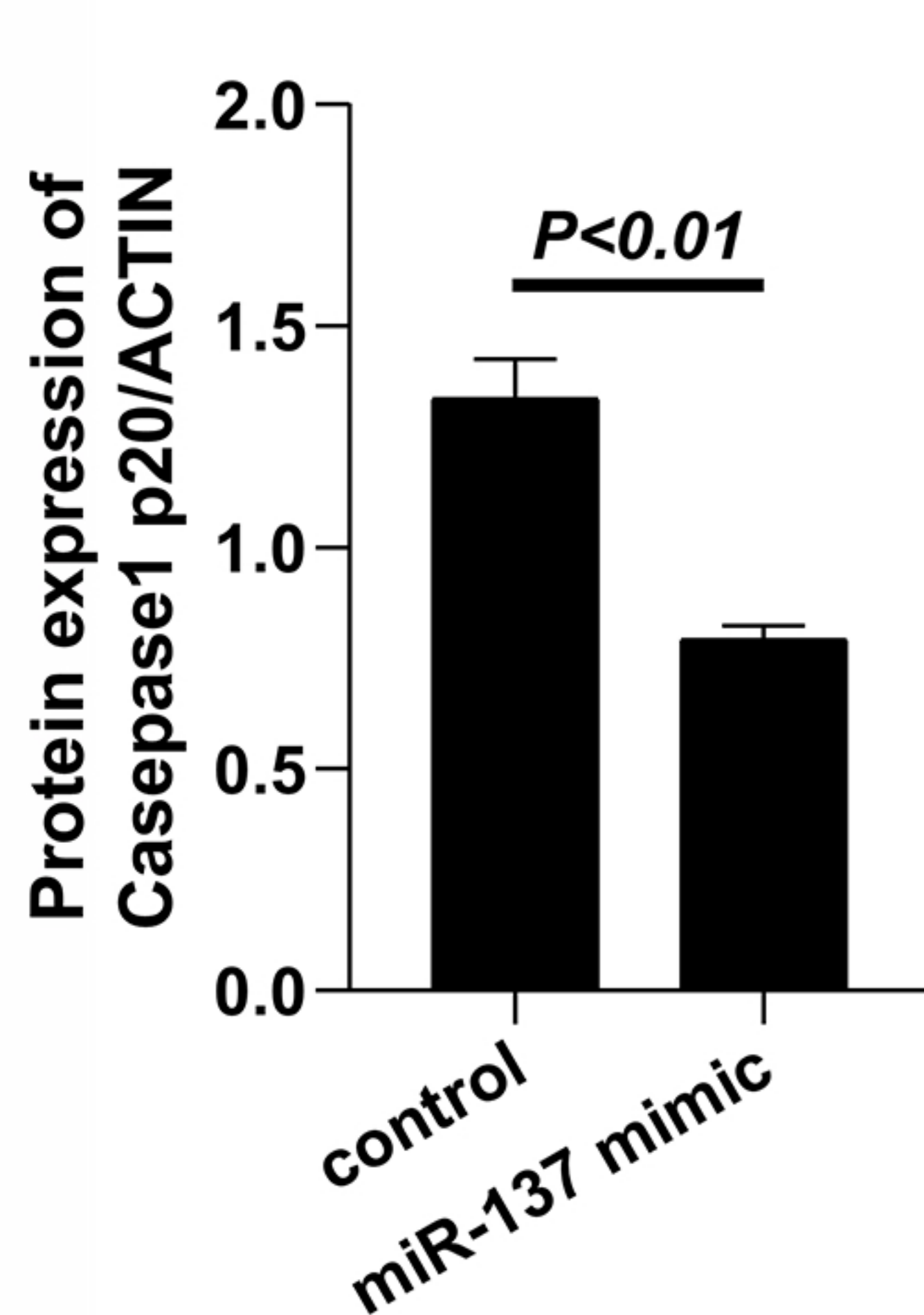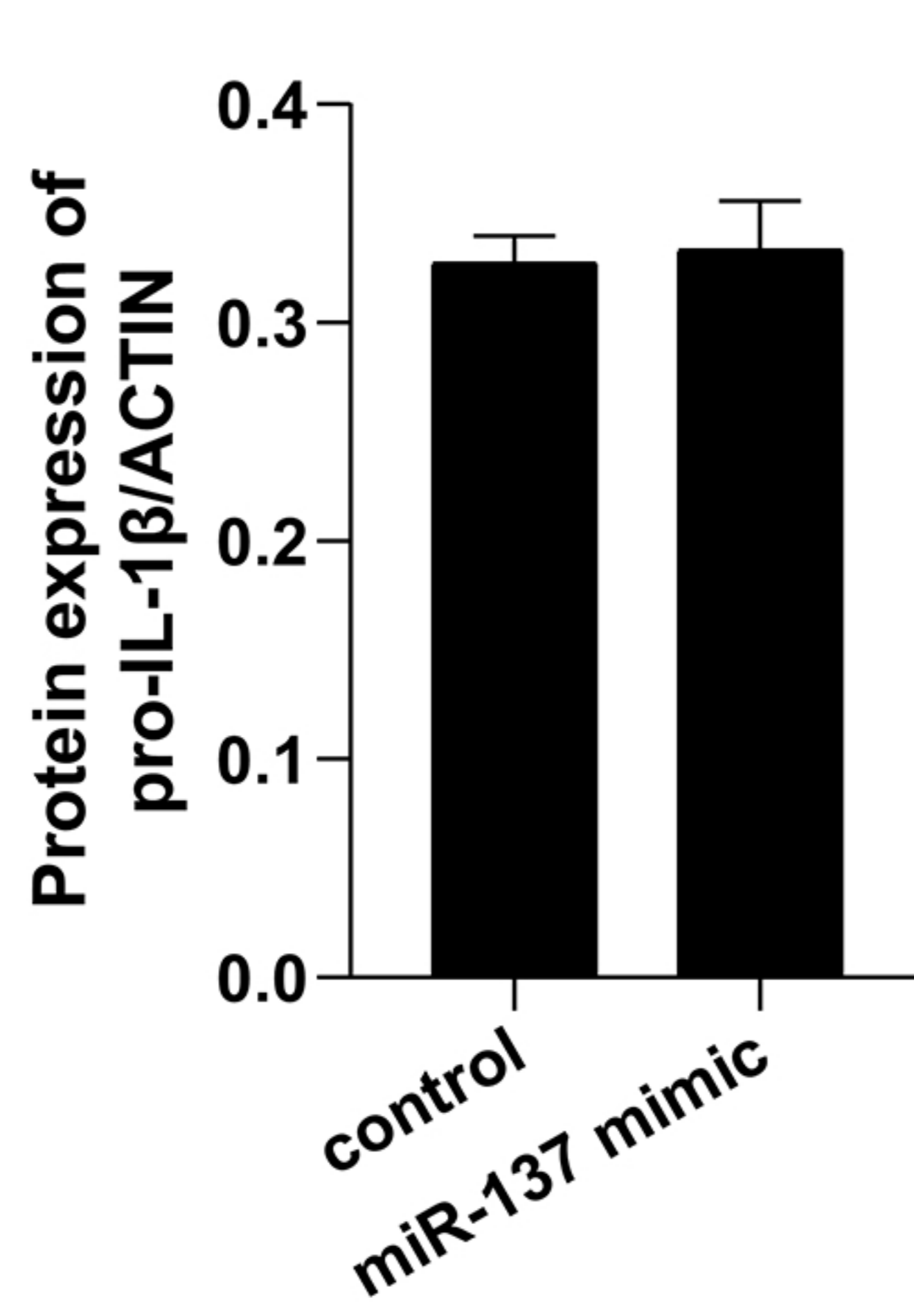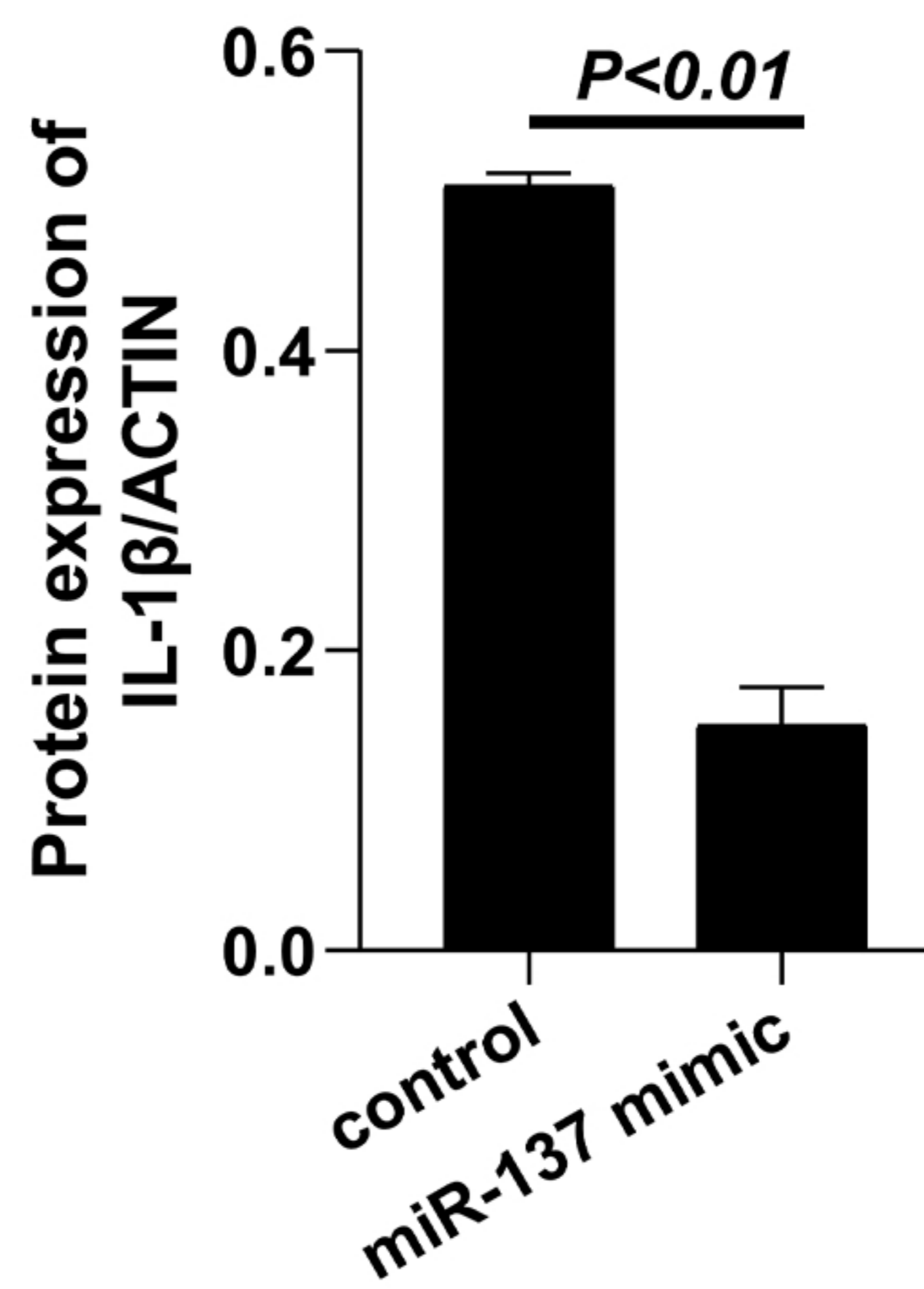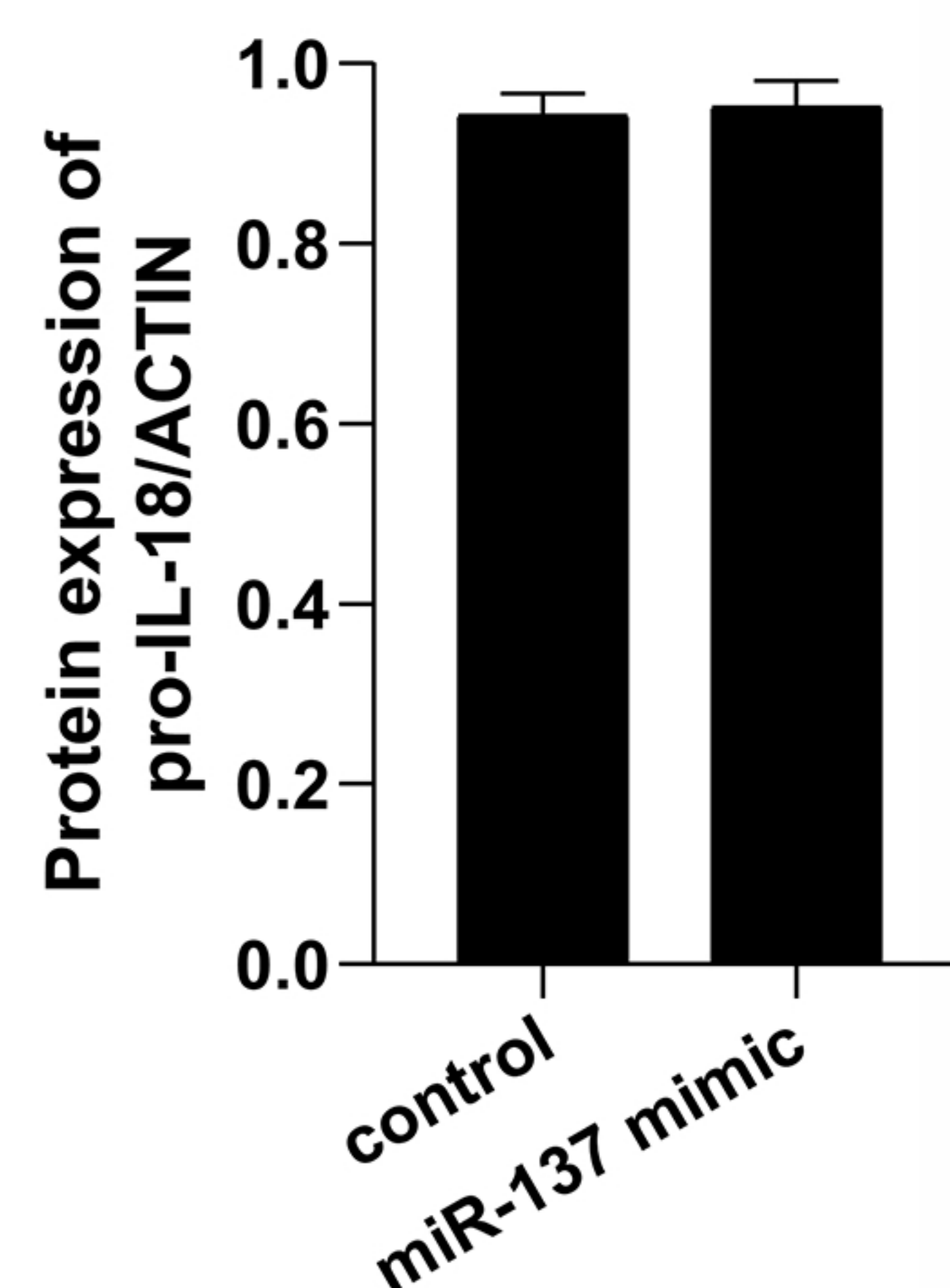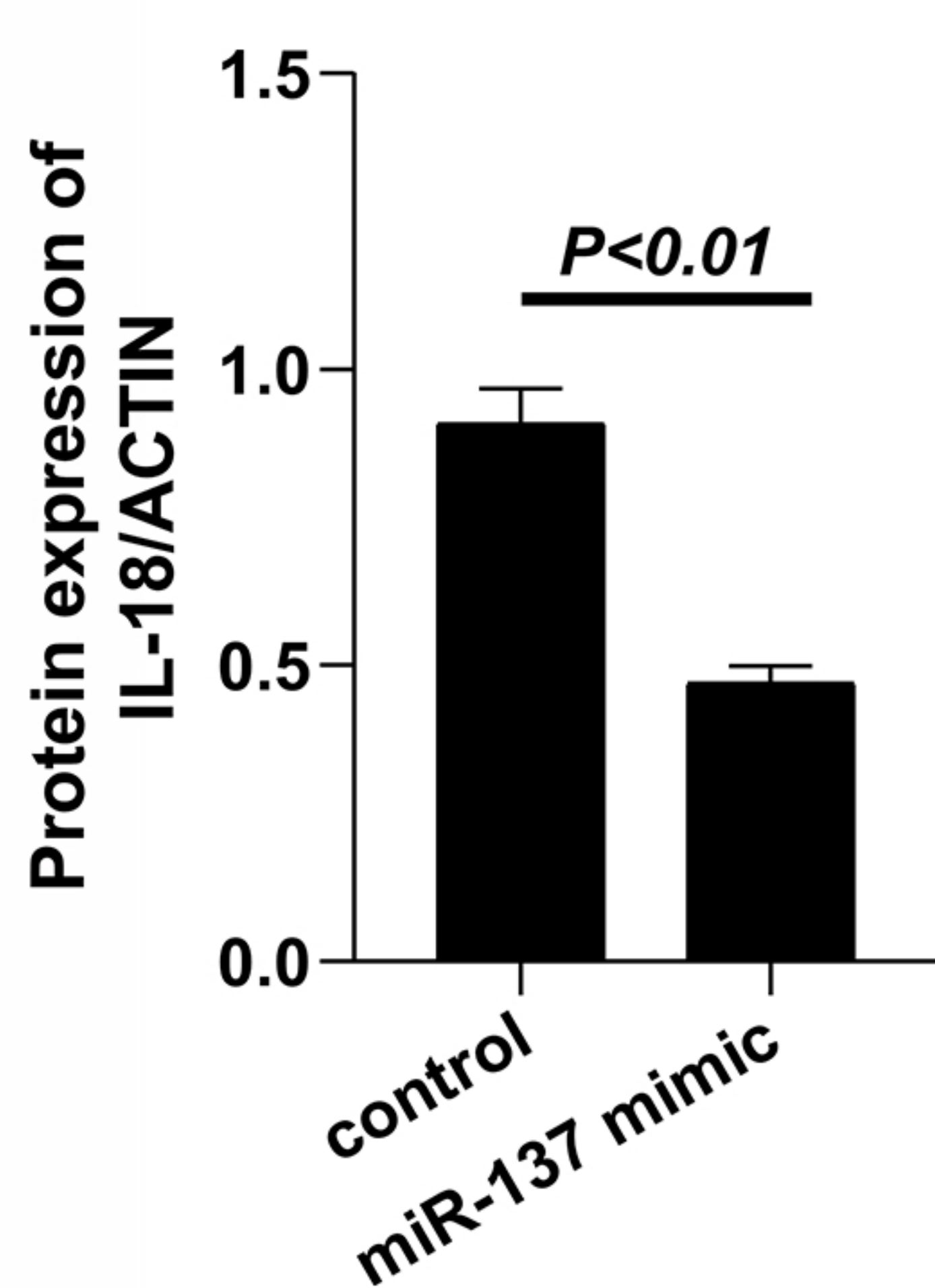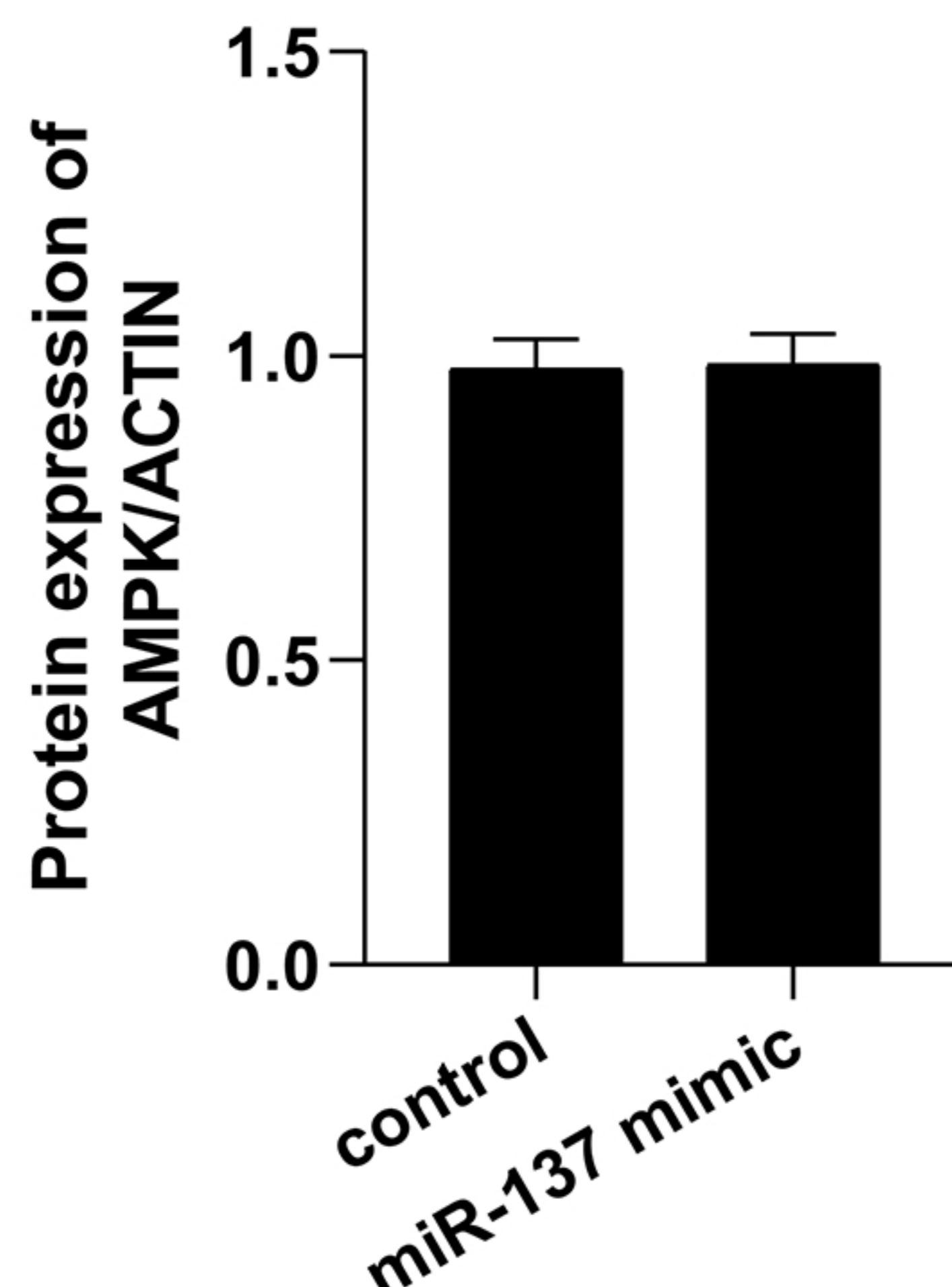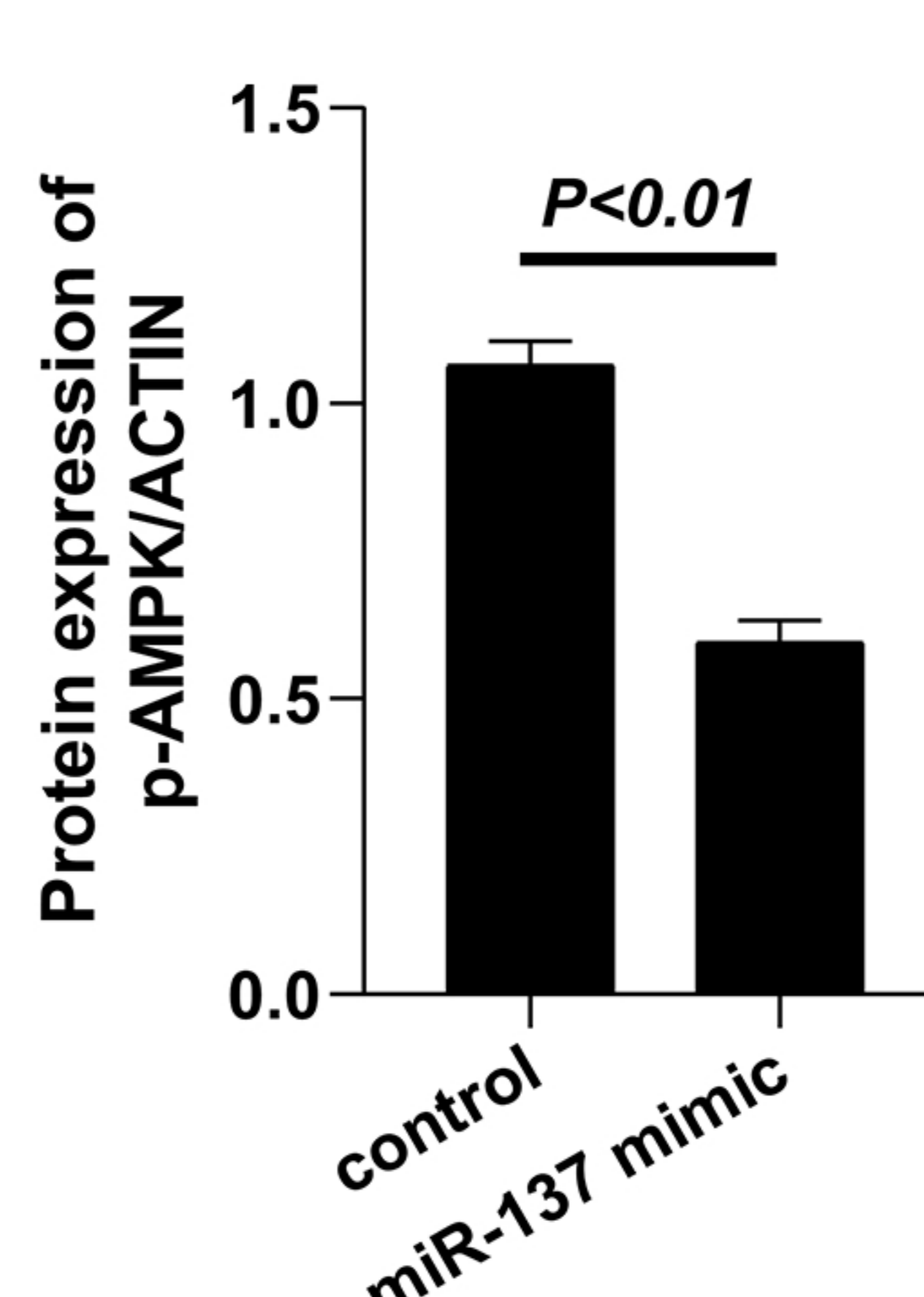

Supplement: Supplementary 3 — Supplementary Figure 3: inflammation and pyroptosis-related protein levels in normal CD4+ T cells after transfected with miR-137 mimic. [file 1241774.f3.pdf]
